# Early-Onset Alcohol Dependence and Multiple Sclerosis: Diagnostic Challenges

**DOI:** 10.3390/ijerph18115588

**Published:** 2021-05-24

**Authors:** Maria Luca, Clara Grazia Chisari, Aurora Zanghì, Francesco Patti

**Affiliations:** Section of Neurosciences, Department “GF. Ingrassia”, University of Catania, Via S. Sofia, 78, 95123 Catania, Italy; lucmaria@tiscali.it (M.L.); clarachisari@yahoo.it (C.G.C.); aurora.zanghi@yahoo.it (A.Z.)

**Keywords:** multiple sclerosis, alcohol dependence, differential diagnosis, diagnostic delay

## Abstract

Multiple sclerosis (MS) is an inflammatory demyelinating disorder characterized by the progressive disruption of the myelin sheath around the nerve fibres. The early initiation of disease-modifying treatments is crucial for preventing disease progression and neurological damage. Unfortunately, a diagnostic delay of several years is not uncommon, particularly in the presence of physical and mental comorbidities. Among psychiatric comorbidities, the role of alcohol misuse is still under debate. In this paper, we discuss a case of early-onset alcohol dependence and its possible role in delaying the initiation of a specific therapy for MS. The differential diagnosis between idiopathic and secondary neurodegenerative disorders is often challenging. When dealing with patients reporting an early-onset substance abuse (likely to present organic damage), clinicians may be prone to formulate a diagnosis of secondary neuropathy, particularly when facing non-specific symptoms. This case report highlights the need for in-depth medical investigations (including imaging) in the presence of neurological signs suggesting a damage of the central nervous system, prompting a differential diagnosis between idiopathic and secondary neurodegenerative conditions. Indeed, a timely diagnosis is crucial for the initiation of specific therapies positively affecting the outcome.

## 1. Introduction

Multiple sclerosis (MS) is an inflammatory demyelinating disorder characterized by the progressive disruption of the myelin sheath around the nerve fibres [1]. Persons with MS can display a variety of symptoms, according to the affected bodily function (e.g., vision, sexuality, movement). Despite this variability, the typical manifestation of MS (relapsing–remitting phenotype) is characterized by relapses followed by periods of recovery [2]. The early initiation of disease-modifying treatments is crucial for preventing disease progression and neurological damage [3]. The diagnosis typically relies upon medical history and neurological examination. If the clinical evaluation is suggestive of MS, an MRI with intravenous contrast agent (gadolinium) is indicated. Cerebrospinal fluid analysis may be performed, along with other exams, such as evoked potentials, optic coherence tomography, blood tests and cognitive tests [4]. Due to the heterogeneity of MS symptoms and variable outcome, the diagnosis is often challenging. To further complicate matters, several cerebrospinal fluid biomarkers have been proposed for diagnosis and prognosis (e.g., oligoclonal bands, oxidative enzymes, IgG index), but only a few are employed in clinical practice [5]. Consequently, a diagnostic delay of several years is not uncommon, particularly in the presence of physical and mental comorbidities [6,7,8]. Among psychiatric co-morbidities, the role of alcohol misuse is still debated. Indeed, literature provides inconsistent data regarding its possible role as a risk factor for MS [9,10]. As a matter of fact, alcohol intoxication, characterized by symptoms that may mimic neurological conditions (e.g., double vision, ataxia, etc.), can represent a confounder when performing the differential diagnosis between idiopathic neurological disorders and alcohol-related neurological impairment [11]. Consistently with this, alcohol use and abuse have been reported to cause a diagnostic delay in amyotrophic lateral sclerosis and stroke [11,12]. Considering MS, the differential diagnosis can be quite challenging and may cause a risky treatment delay. Overall, the MRI findings, along with a careful anamnesis, acquire a fundamental importance in the decision-making phase [13]. In the following report, we discuss a case of early-onset alcohol dependence that may have delayed the diagnosis of MS.

## 2. Case Report

A female patient in her thirties, suffering from alcohol dependence, was admitted to the psychiatric ward of our University Hospital “Policlinico-San Marco” of Catania in 2020 for suspected alcohol abstinence syndrome, having stopped drinking in the previous 3 days. At admission, she showed a condition of hyperarousal, visive hallucinations, tremors and gait difficulty.

Her blood tests showed macrocytic anemia and electrolyte imbalance. The liver ultrasound detected “markedly enlarged liver with thickened and hyperechogenic echo-structure, compatible with chronic hepatopathy”.

The discharge documents from a previous hospitalization (year 2017, diagnosis of “epileptic seizure during alcohol abstinence”) reported the suspicion of an inflammatory demyelinating disorder, due to a brain and spine MRI report: “numerous areas of altered signal at the periventricular site, some of them confluent. No contrast-enhancing lesions observed. Considering the spine, a single lesion at C3 level has been observed with no contrast enhancement”. The patient stated that these lesions had been explained to her as possibly secondary to alcoholism. As a result, she did not seek further advice.

In light of the MRI report, our MS centre was required to provide a neurological consultation.

At the neurological visit, the patient complained fatigue and numbness of her toes (for the past two months) and reduced visual acuity at the right eye (since 2016). The anamnesis unveiled an early-onset alcohol misuse. Indeed, the patient approached alcohol at the age of 13, when she and her friends managed to buy a cocktail despite being underage. She found alcohol really “tasteful”. Hence, after episodes of binge drinking with her peers, she started buying wine to drink alone and in secret. The problem was noticed by her parents, who prompted her to seek help as an outpatient. However, her compliance was poor. She did not take the psychopharmacological treatment (unknown) prescribed by psychiatrists working in centres for addiction and she did not show up at the follow-up visits. The main reasons for drinking were, according to her experience, alcohol’s good taste and power to give her “energy to do things and chores”. Moreover, since she was able to work (even if intermittently) and have a social life, she did not consider her condition as a pathological one. The years passed, and she was never able to interrupt alcohol consumption for more than 14 days. Before the abrupt withdrawal determining her admission to the psychiatric ward, she was consuming an average of three litres of strong beer/per day, with episodic use of spirits. She used to store a large amount of bottles in her house (in several “hiding places”). When drinking, she completely overlooked her diet, not feeling the need to eat at all. She smokes 15–20 cigarettes/per day and she does not abuse of other substances.

In her medical history:

(1) Two falls due to postural instability (difficulty in maintaining her balance).
(2) A first hospitalization in a private clinic for “alcoholism” about six years ago. She did not follow the medical advice after discharge.
(3) In 2016, optic neuritis characterized by an acute reduction of visual acuity (right eye), without pain. She refers to having received a diagnosis of “neuropathy secondary to alcohol abuse” (no documentation shown). She was administered a course of oral steroids (1 mg/kg) for 10 days, but recovery was poor: the deficit of visual acuity is still present.
(4) In 2017, hospitalization for epileptic seizure. In this occasion, she underwent her first MRI that showed inflammatory-demyelinating lesions (reported above).

When asked about her physical symptoms in her lifetime, apart from the episode of optic neuritis (and the recently occurring fatigue and numbness of the toes) she reported nothing more than postural instability (for several years, even when sober).

**Figure 1:**
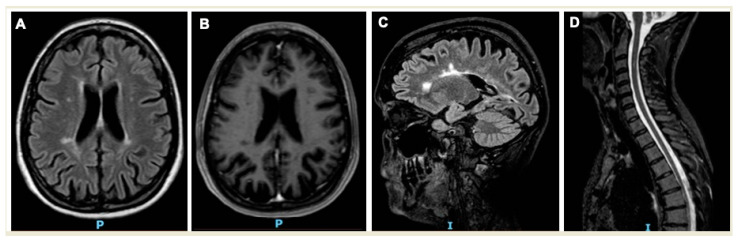
Brain and spine MRI at diagnosis of MS. Legend: the figure shows four MRI sequences (**A**–**D**). Explanation below. (**A**) Axial T2-weighted using a fluid attenuated inversion recovery (FLAIR), sequences of the brain MRI show typical periventricular hyperintense lesions due to the demyelination of the white matter. (**B**) The hyperintense lesions observed in sequence A appear hypointense in axial T1-weighted sequences of the brain MRI, due to axonal destruction and irreversible damage (also called “black holes”). (**C**) Sagittal T2-weighted using a fluid attenuated inversion recovery (FLAIR), sequences of the brain MRI show periventricular hyperintense lesions along the axis of the medullary veins, perpendicular to the body of the lateral ventricles and of the callosal junction (also called “Dawson’s fingers”). (**D**) Sagittal T2-weighted sequences of the spine MRI show a hyperintense demyelinating lesion at C3 level.

The neurological examination, performed during the consultation, showed ataxic gait and hyperreflexia on the four limbs. The maximal acuity of the worst eye was of 20/60. The Expanded Disability Status Scale (EDSS) score was 2.5 (mild disability, preserved ambulation). A new brain MRI exam, performed during hospitalization (Figure 1) and revised by an expert neurologist, showed: “in the long TR sequences, some millimetric lesions located in the paratrigonal white matter, in the nucleocapsular site, at the semi-oval centres on both sides. Some of the aforementioned lesions are radially positioned with respect to the lateral ventricles”.

Therefore, a diagnosis of relapsing–remitting MS with sequelae was formulated, according to the 2017 revisions of the McDonald criteria by Thompson et al. [14]. A specific therapy with “fingolimod” was planned. The drug, approved by the European Medicines Agency as a treatment for relapsing–remitting MS, has been demonstrated to be efficient in terms of relapse rates and outcome [15]. The clinician’s choice fell upon fingolimod because the latter is indicated as a first-line therapy in patients naïve to treatment and with highly active MS [16]. The patient started the therapy after discharge. The follow-up of the patient will include another neurological examination, evoked potentials and another MRI (to be performed1 year after diagnosis, as per clinical practice).

## 3. Discussion

**Figure 2:**
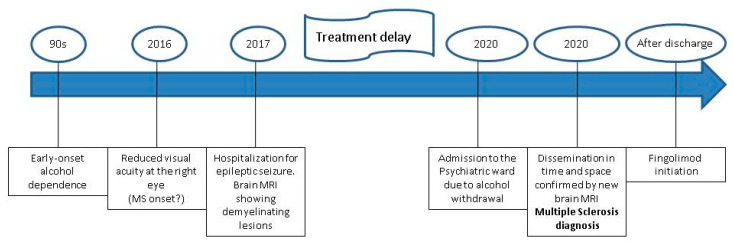
Timeline. The Figure shows the chronological summary of the most salient events in the medical history of the patient. Please note the significant diagnostic delay from the first MRI to the actual diagnosis of multiple sclerosis.

**Figure 3:**
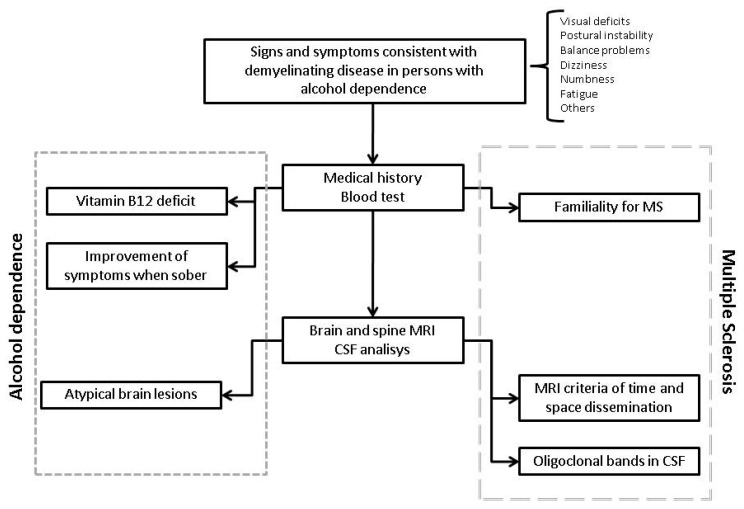
Alcohol-related neurological condition or multiple sclerosis? The figure shows (in an oversimplified manner) our reasoning when handling this case. The flowchart has been enriched with general data (e.g., familiarity, cerebrospinal fluid analysis) that may be useful in similar cases. The left side of the figure summarizes the aspects that may suggest a diagnosis of neurological condition secondary to alcohol dependence. The right side shows the aspects soliciting a diagnosis of multiple sclerosis. CSF: cerebrospinal fluid.

The diagnosis of MS was guided by the 2016 MAGNIMS MRI criteria of dissemination in space (involvement of at least two of the following brain areas: periventricular, cortical-juxtacortical, infratentorial, spinal cord, optic nerve) and time (a new lesion when compared to a previous scan, T2 bright lesion and/or gadolinium-enhancing, presence of enhancing lesion and a non-enhancing T2 bright lesion on any one scan) [17]. The medical history of this patient (summarized in Figure 2) shows how alcohol dependence may have added complexity to the diagnosis of MS, ultimately delaying it. As a matter of fact, during recent years, the patient only showed postural instability. Despite MS typically impairing postural control [18], instability is a non-specific symptom, also being frequently reported among alcohol-dependent individuals [19]. Indeed, excessive sway during quiet standing is a typical feature of persons with alcohol abuse, that often persists even after long periods of sobriety [19]. The episode of optic neuritis could have been the turning point to reach a diagnosis of MS. Indeed, optic neuritis (namely, the inflammation of the optic nerve affecting vision) is the presenting feature of MS in up to 20% of patients [20,21]. Moreover, it has been estimated that half of the patients diagnosed with MS will experience an episode of optic neuritis during the disease course [20]. Notwithstanding, it cannot be denied that optic neuropathy is not uncommon among alcohol-dependent individuals [22]. The main cause of damage of the optic nerve in these patients lies in their typical condition of malnourishment. Indeed, patients with alcoholism often show reduced levels of folate and B-complex vitamins, whose deficiency determines the accumulation of neurotoxic substances, ultimately damaging the optic nerve [23]. As a result, once again, the overlapping features between alcohol dependence and MS may have “masked” the concomitant diagnosis of MS. Unfortunately, the lack of documentation relating to the episode of optic neuritis does not allow us to verify whether the levels of folate and B-complex vitamins were analyzed at the time. The patient’s reduced interest in her own health whilst struggling with a decades-long addiction, further delayed the initiation of a specific therapy for MS from 2017 (suggestive MRI) to 2020. To conclude on a positive note, at the time of the in-depth interview to clarify and verify the information reported in this case report, the patient was sober for 100 days, showed good compliance in relation to her psychiatric medication and was about to undergo a job interview. This case report has some limitations. Firstly, the diagnostic process could have been enriched by further evaluations (e.g., cerebrospinal fluid analysis, evoked potentials), that have not been performed due to the COVID-19 pandemic. Secondly, the patient did not provide any documentation regarding the optic neuritis. However, the medical history, along with the clinical and radiological data, guided the diagnosis of MS, as detailed above and illustrated in Figure 3.

## 4. Conclusions

The differential diagnosis between idiopathic and secondary neurodegenerative disorders is often challenging. When dealing with patients reporting an early-onset substance abuse (likely to present organic damage), clinicians may be prone to formulate a diagnosis of secondary neuropathy, particularly when facing non-specific symptoms. This case report highlights the need for in-depth medical investigations (including imaging) in the presence of neurological signs prompting a differential diagnosis between idiopathic and secondary neurodegenerative conditions. Indeed, a timely diagnosis is crucial for the initiation of specific therapies positively affecting the outcome.

## Data Availability

This paper does not contain statistical data, but only clinical ones, retrieved from the patient’s medical chart. Hence, for confidentiality reasons, the sources of the data are not publicly available.
